# Identifying candidate genes and biological pathways in muscle development through multi-tissue transcriptome comparisons between male and female geese

**DOI:** 10.1038/s41598-024-67560-2

**Published:** 2024-07-16

**Authors:** Yunzhou Yang, Cui Wang, Shufang Chen, Yi Liu, Huiyan Jia, Huiying Wang, Daqian He

**Affiliations:** 1 Institute of Animal Husbandry and Veterinary Science, Shanghai Academy of Agricultural Science, Shanghai, 201106 China; 2 Institute of Animal and Poultry Science, Ningbo Academy of Agricultural Sciences, Ningbo, 315040 China

**Keywords:** Geese, Gender effects, Growth, Muscle fiber, Bone development, Calcium, Computational biology and bioinformatics, Genetics

## Abstract

Males and females have long shown disparities in body weight and height; yet, the underlying mechanisms influencing growth and development remain unclear. Male and female Zhedong White Geese (ZDW) geese have long been selected for large body size and egg production, respectively. This led to a large difference in body weight between males and females, making them a unique model for studying the effects of sex on growth and development. This study aimed to elucidate these mechanisms by comparing the transcriptomes of muscle and pituitary tissues in male and female ZDW geese to identify the critical genes responsible for the effects of sex on growth performance. Our analysis revealed 1101 differentially expressed genes (DEGs) in leg musculature (507 upregulated, 594 downregulated), 773 DEGs in breast musculature (311 upregulated, 462 downregulated), and 517 DEGs in the pituitary gland (281 upregulated, 236 downregulated) between male and female geese. These DEGs were significantly enriched in gene ontology and Kyoto Encyclopedia of Genes and Genomes (KEGG) pathways associated with endocrine metabolism (e.g., hormonal activities), muscle formation (e.g., sarcomere and myofibril), and bone formation (e.g., bone morphogenesis and cartilage formation). The upregulated genes in males were enriched in KEGG pathways involving nutrient digestion and absorption (vitamin and protein), as well as the secretion of digestive juices (gastric acid and bile). Through protein–protein interaction analyses, we also observed high-density gene networks related to muscle fiber development, calcium ion metabolism, mitochondrial respiratory chain, and bone development. Therefore, our multi-tissue transcriptome analysis provides a deeper understanding of the complex and systematic gender-driven effects on growth and development in geese. *IGF1*, *GHRHR*, and *NCAPG*-*LCORL* and pathways related to myogenesis might play vital roles in gender differences before hormones exert their effect.

## Introduction

Intrasexual selection has exerted intensive selection pressure on both males and females in several species^1^. In most cases, males have to fight for more mating opportunities, whereas females compete for more reproductive resources differently^2^. Studies have claimed that intrasexual selection acts mainly on males. As a result, it has been commonly observed that males evolve to grow faster and are heavier and better-armed across several species^3^. These visible phenotypic differences due to the selection of genders were just the tip of the iceberg for sexual dimorphisms. The terra incognita of gender impacts on growth performance has been widely discussed and studied over the last few decades^4–7^. Numerous studies have been undertaken to understand the intricate mechanistic foundation of gender disparities in growth and development. The primary and pivotal factor causing gender distinctions across various domains is the variation of hormone categories and concentrations. The anterior pituitary is a central gland that synthesizes and secretes growth hormone (GH), adrenocorticotropic hormone (ACTH), and thyroid stimulating hormone (TSH)^8^. The functions and effects of these hormones on growth performance have been investigated in domesticated animals. Treating calves with GH at different concentrations led to significantly higher carcass body weights^9^, whereas disruptions of the GH-IGF1 axis caused dwarfism^10^. However, the effects of gender on growth and development often involve a complicated process, which is difficult to conclude based on a single piece of evidence. In broilers, males showed higher growth performance, reflected in weekly body weight growth, than females. This superiority manifests across multiple facets, including increased feed consumption, enhanced feed conversion ratio, and a greater abundance of muscle fibers^7,11^. This suggests that more information and studies are required to understand this phenomenon.

Given the wide physiological differences and profound effects on performance associated with gender, it is crucial to incorporate this factor into a model for exploring the intricate associations between genotypes and phenotypes^12,13^. However, the avoidance of this factor has led to an incomplete understanding of the basic mechanisms governing the effects of gender. This study concentrates only on the effects of gender on the growth and development of geese and aims to elucidate the underlying mechanisms of these effects. Variances in muscle mass and hormones secreted by the pituitary at either phenotypic level or molecular level are known to affect body weight and hormone concentrations^14,15^. ZDW goose has been always kept by villagers to guard their yard as geese were vigilant and noisy and male geese were selected for larger body size than females while females were selected for reproduction performance. Due to precocious sexual maturity and large difference in growth performance between male and female geese, the ZDW goose breed was selected in our study^13^. By performing one-way (the factor of sex) comparative transcriptomic analyses, we aimed to detect critical genes leading to differences in growth performance between male and female geese. These findings will provide valuable insights at the transcriptional level, enhance our understanding of the effects of sex on growth performance, and identify candidate genes that can be used as targets in molecular-assisted selection for body weight control.

## Methods

All animal protocols were conducted according to standard guidelines and regulations and were approved by the Science and Technology Commission of Shanghai Municipality (STCSM) under license number SYXK (HU): 2015–0007. Our guidelines were also in accordance with ARRIVE guidelines (https://arriveguidelines.org).

### Samples

A total of 500 Zhedong White (ZDW) geese (male: 250; female: 250) were selected after identifying their sex by observing the morphology of the anal vent. These geese were hatched on the same day and raised in the same farming house. Throughout our experiments, the geese were provided with unlimited feed and water. The body weights of the geese were recorded at 0, 14, 28, 42, 56, and 70 days. Body weight at the age of 70 days (BW70) is an important quantitative trait under intensive selection and is widely used in goose breeding because it reflects the growth performance of geese. Four male and four female geese at the age of 70 days were selected randomly for subsequent sampling. Leg muscle, breast muscle, and pituitary tissues were collected and immediately frozen in liquid nitrogen. For histological experiments, muscles from the leg and breast were excised and preserved in neutral paraformaldehyde fixative.

### Measurement of blood biochemistry indices

After coagulation of the blood samples in a 10 mL glass tube without anticoagulants, 2 mL of serum was collected and preserved in a − 20 °C freezer until subsequent use. The biochemistry indices of glucose (Glu), triglycerol (TG), cholesterol (CHO), high-density lipoproteins (HDL), and low-density lipoprotein (LDL) were determined using specific kits S03039, S03027, S03042, S03025, and S03029. Concentrations of estradiol (E2), testosterone (T), and GH were determined using ELISA. Kits used for the specific index tests and the automatic biochemistry analyzer were all obtained from Rayto (Shenzhen, China).

### HE staining for leg muscles among female and male geese

Histology studies were performed at a commercial company (ServiceBio, China). Leg muscles were collected and quickly immersed in neutral paraformaldehyde fixative. The corresponding tissue sections were prepared according to the experimental protocol of ServiceBio, including fixation, embedding, paraffin section, frozen section, and other processes. Paraffin sections were further processed by dewaxing and hydration in environmentally friendly dewaxing transparent liquid I and II for 20 min each, followed by treatment with anhydrous ethanol I and II for 5 min each, 75% ethyl alcohol for 5 min, and finally rinsed with tap water. The sections were then frozen and thawed to room temperature, followed by fixation in tissue fixing solution for 15 min, and finally rinsed with running water. After treatment with HD constant staining pretreatment solution for 1 min, the sections were stained in hematoxylin solution for 5 min, rinsed with tap water, and then stained with eosin for 15 s. The dehydration and sealing steps were performed in the following reagents sequentially: absolute ethanol I (2 min), absolute ethanol II (2 min), absolute ethanol III (2 min), normal butanol I (2 min), normal butanol II (2 min), xylene I (2 min), xylene II (2 min), and finally sealing with neutral gum. The sections were then observed and analyzed using the software CaseViewer (version 2.4, 3DHISTECH, Hungary). The nucleus was stained blue, and the cytoplasm was stained red.

### Transcriptome sequencing of tissues from male and female ZDW geese

Muscles and pituitary tissues were collected from slaughtered geese and quickly stored in liquid nitrogen. Frozen samples were ground in liquid nitrogen and dissociated in TRIzol agent (Invitrogen, USA). Thereafter, chloroform, isopropanol, and 75% ethanol were used for lysis, RNA precipitation, and purification, respectively. The RNA integrity (RIN) was determined using fragment analyzer system (Agilent 5400, USA). RIN values of all sample RNAs ranged between 8.4 and 9.3, indicating that the extracted RNAs were of high quality. Libraries prepared using freshly extracted RNA were sequenced on the Illumina NovaSeq 6000 platform (Personal Biotech, Shanghai). Each sample was sequenced with 20 million read pairs, covering 6 GB of raw data. The quality of sequenced reads was evaluated using the software FastQC (V0.11.9, https://www.bioinformatics.babraham.ac.uk/projects/fastqc/). The reads with low quality, as well as adapters, were trimmed using the software Cutadapt (V1.15, https://cutadapt.readthedocs.io/en/stable/))^16^. The software STAR (V2.7.10a, https://github.com/alexdobin/STAR), featureCounts (V2.0.6, https://subread.sourceforge.net/), and edgeR (V3.32.1, https://bioconductor.org/packages/release/bioc/html/edgeR.html) were used to align reads to the reference genome (GCF_002166845.1, Sichuan White geese (SCW), *Anser cygnoides*) and detect differentially expressed genes (DEGs) between male and female geese^17–20^. The significance threshold was absolute values of log_2_(fold changes) of > 1 and *P*-value of < 0.05. Goseq software was used for GO and signaling pathway enrichments (V1.42.0, https://new.bioconductor.org/packages/release/bioc/html/goseq.html)^21^. Due to poor information on the goose GO and signaling pathways, gene symbols from the goose assembly (GCF_002166845.1) were first transformed to human gene symbols, as they can be applied in the human GO/KEGG information deposited in GOseq^22,23^. Selecting the chicken genome as the reference assembly for read alignment and functional analyses was also feasible, but several DEGs, GO terms, and pathways were excluded. Using a human database will also miss DEGs or GO terms, but the associated cost is much lower than that of using other genomes.

### Construction of protein–protein interaction network

The upregulated genes detected in pituitary tissues between males and females were used as seed genes in the STRING server (https://cn.string-db.org/), and a tab-separated tsv format file containing interaction relationships could be created and downloaded. This tsv file contained interactions among different genes and was the input file for Cytoscape software (V3.10.2, https://cytoscape.org/)^24^. Users could customize the network style and select reliable node values.

### Validation of candidate DEGs by real-time quantitative PCR

The first-strand cDNA was synthesized from total RNAs using a TransScript-All-in-One kit (gTransgen, China). β-actin was used as the reference gene, and the primers reported for this gene in our previous study were synthesized in Sangon (Shanghai, China)^25^. Primers for twelve DEGs (*GHRHR*, *TMEM163*, *TYRP1*, *ANO4*, *SYT4*, *KRT222*, *POU1F1*, *HOXA5*, *IGFN, APOB, BMP6,* and *IGF1*) were designed using online tools available on NCBI (Table S9). qPCR experiments were performed using Quant 5.0 (Roche) and the RT-PCR kit from TAKARA (RR066A, Dalian, China), according to the manufacturer’s instructions.

## Results

### Weekly trend in body weight among male and female ZDW geese

At the hatching stage, female ZDW geese showed higher body weight (BW0) than males (*P* < 0.05). Starting from 14 days of age, male geese consistently displayed significantly higher body weight than females (*P* < 0.01). The difference between the body weights of male and female geese increased with age. The male geese were 4.16% and 13.73% heavier than females at BW14 and BW70, respectively.

### Blood biochemistry indices (BCIs) among ZDW geese at the age of 10 weeks

Among all five BCIs, only the blood glucose (GLU) concentration was significantly higher in males than in females (*P* < 0.05). Although males also showed higher TG concentrations, the difference was not statistically significant. For the BCIs of CHO, HDL, and LDL, females displayed higher levels than males, although the differences were not statistically significant (*P* > 0.05).

### Hormone concentrations among ZDW geese at the age of 10 weeks

The concentrations of three hormones in male and female geese were compared. The concentration of E2 was higher in females than in males, whereas those of T and GH were slightly higher in males. However, no significant differences were observed in hormone concentrations between female and male geese (*P* > 0.05).

### Histological investigations of muscle fiber properties between male and female geese

Transverse sections and longitudinal sections of male and female leg muscles were stained by hematoxylin and eosin. Areas of transverse sections for each muscle fiber were marked manually and calculated using the software CaseViewer (V2.4, 3DHISTECH, Hungary).

**Figure 1:**
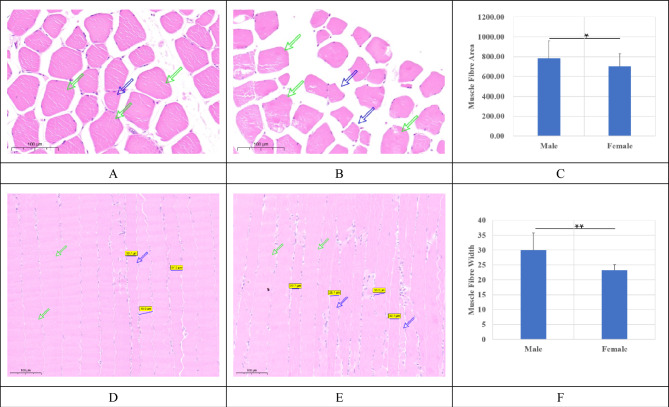
Histological staining for leg muscles from male and female geese. (**A**) and (**D**) were from male geese while (**B**) and (**E**) were for female geese. A/B and D/E were transections and longisections, respectively. The black horizontal lines in A/B/D/E were the 50 μm plotting scales. (**C**) were the areas of muscle fibers from the cross sections (μm^2^) between male and female geese. (**D**) were the width for the leg muscle fibers from longisections (μm) between male and female geese.

Although some male muscle fibers were smaller and thinner (blue arrows in Fig. 1A,D) than those of females, collectively counting the fibers in different fields under the microscope revealed that the areas of leg muscle fibers from male geese were significantly higher than those from female geese (*P* < 0.05, Fig. 1A–C). To confirm these findings, longitudinal sections of muscles were also investigated. The widths of leg muscle fibers from these sections were significantly higher in male than in female geese (*P* < 0.01).

### DEGs in tissues between male and female ZDW geese at the age of 10 weeks

**Figure 2:**
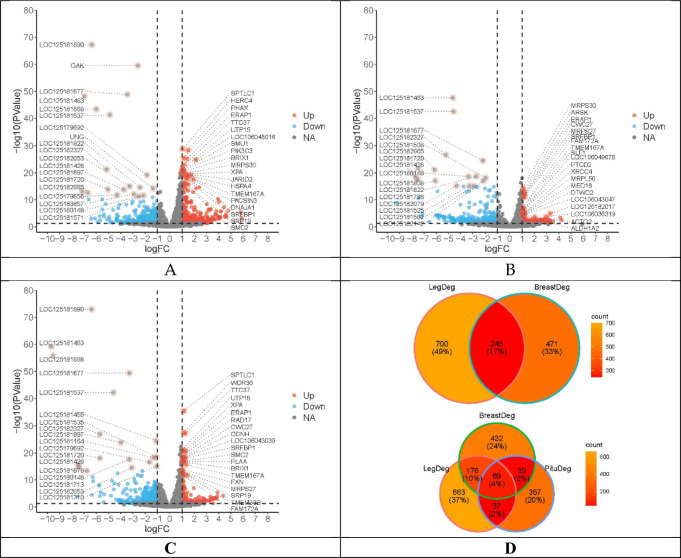
Volcano plots displayed differentially expressed genes (DEGs) between different comparative transcriptome analyses: (**A**–**C**) were DEGs from leg transcriptomes (male vs female), breast muscle transcriptomes (male vs female), and pituitary transcriptomes (male vs female). (**D**) was the number of common DEGs (cDEGs) among different groups.

Male and female geese at age of ten weeks were selected because geese showed large difference at the time (Male: 4371.71 ± 399.58; Female: 3771.14 ± 389.11, Table 1). Further, the age of 10 weeks is the time point at which farmers begin to select geese for breeding in next generation and BW10 reflects individual’s growth performance. Breast muscles, leg muscles, and pituitary tissues were selected from eight samples (male: 4; female: 4) for subsequent library construction and transcriptome sequencing. After filtration of the raw data, each sample was covered by more than 20 million read pairs (20,196,145.04 ± 774,767.23). The mean depth was 5.41 (± 0.21). For the leg, breast, and pituitary tissues, 1101 (507 upregulated; 594 downregulated), 773 (311 upregulated; 462 downregulated), and 517 (281 upregulated; 236 downregulated) DEGs were identified in males compared with females (*P* < 0.01), respectively. Of these DEGs, 245 were common between leg and breast muscle transcriptomes from male and female samples, including *IGFALS* and *NCAPG*, among others (Fig. 2A). However, only 99 out of 245 common DEGs were annotated well, whereas the remaining 146 genes did not show clear gene information. More gene information is listed in Tables S1–S3.

**Table 1:** Body weight (g) of male and female ZDW at different ages.

Genders	BW0	BW14	BW28	BW42	BW56	BW70
Male	105.44 ± 10.78^a^	465.69 ± 66.67^A^	881.21 ± 140.37^A^	2023.51 ± 250.8^A^	3444.85 ± 437.45^A^	4371.71 ± 399.58^A^
Female	107.77 ± 7.34^b^	446.33 ± 68.69^B^	813.04 ± 131.29^B^	1847.46 ± 316.81^B^	3062.69 ± 310.91^B^	3771.14 ± 389.11^B^

(1) BW is short for body weight. BW0 refer to the body weight when hatched. BW14/28/42/56/70 are the body weight at 14/28/42/56/70 days since hatched. (2) In the same column, different lower-case letters indicated the significance level of 0.05 and upper case for 0.01.

Of the 245 DEGs, 69 were also present in the comparative transcriptome analysis for all three tissue types between male and female geese, including *MRPS27*, *MRPPS30*, and *SLC2A9*, among others (Fig. 2B). More genes are listed in Tables S1–S3.

### Gene ontology terms (GOs) enriched by DEGs

DEGs between male and female leg, breast muscles, and pituitary tissues were enriched in 1043, 622, and 1068 GO terms, respectively (Tables S4–S6). GOs enriched by DEGs from leg muscle transcriptomes showed hormonal activity (GO: 0,005,179). A total of 109 common GO terms were detected by both DEGs from leg and breast muscle transcriptomes, covering functions such as vitamin transport (GO: 0,051,180), glucose transmembrane transporter activity (GO: 0,005,355), and lipid transport involved in lipid storage (GO: 0,010,877, Tables S5–S7).

**Figure 3:**
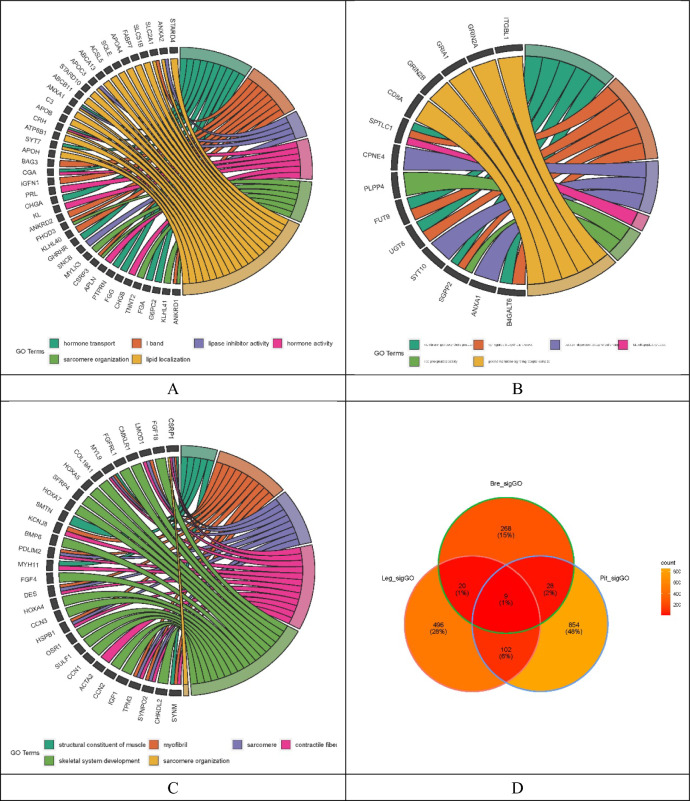
Gene Ontology enrichment analysis using DEG between leg muscle (A), breast muscle (B) and pituitary tissues (C). Circular plots display six GO terms. (D) showed common GOs detected in all three different tissues. Other significantly enriched GO terms were listed in Table S4-S6.

Several GO terms enriched by DEGs in the pituitary transcriptomes were related to muscle development, such as structural consistency of muscle (GO: 0,008,307), sarcomere (GO: 0,030,017), myofibril (GO: 0,030,016), and contractile fiber (GO: 0,043,292), among others*.* GOs related to bone development and hormone metabolism, including bone mineralization (GO: 0,030,282), bone growth (GO: 0,098,868), hormone activity (GO: 0,005,179), and response to steroid hormone (GO: 0,048,545), were also detected (Fig. 3). More detected GO terms are listed in Tables S4–S6.

### KEGG pathway enrichment analyses

**Figure 4:**
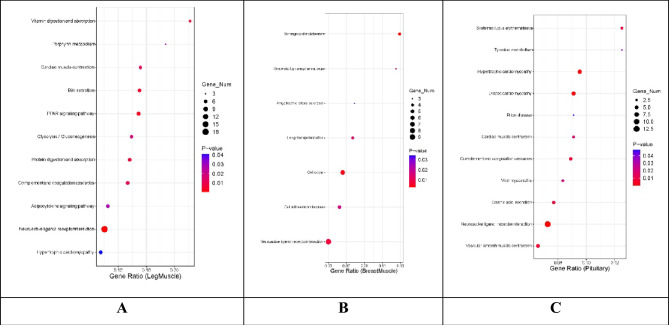
The most significantly enriched KEGG pathways enriched using differentially expressed genes between male and female muscle and pituitary tissues. (**A**) Leg muscle; (**B**) Breast muscle; (**C**) Pituitary.

Based on upregulated DEGs in male geese, 11, 7, and 11 pathways were enriched in leg muscle, breast muscle, and pituitary tissues, respectively (Fig. 4). In leg muscles, 11 pathways were significantly enriched in males, including vitamin digestion and absorption, cardiac muscle contraction, and bile secretion*.* Breast muscles showed significant enrichment in seven tested pathways, including neuroactive ligand-receptor interaction. In pituitary tissues, significant enrichment was observed in 11 signaling pathways, including cardiac muscle contraction, hypertrophic cardiomyopathy, and gastric acid secretion, among others*.* The neuroactive ligand-receptor interaction pathway was consistently detected in all three tissues of male geese.

### Protein–protein interaction (PPI) analysis

**Figure 5:**
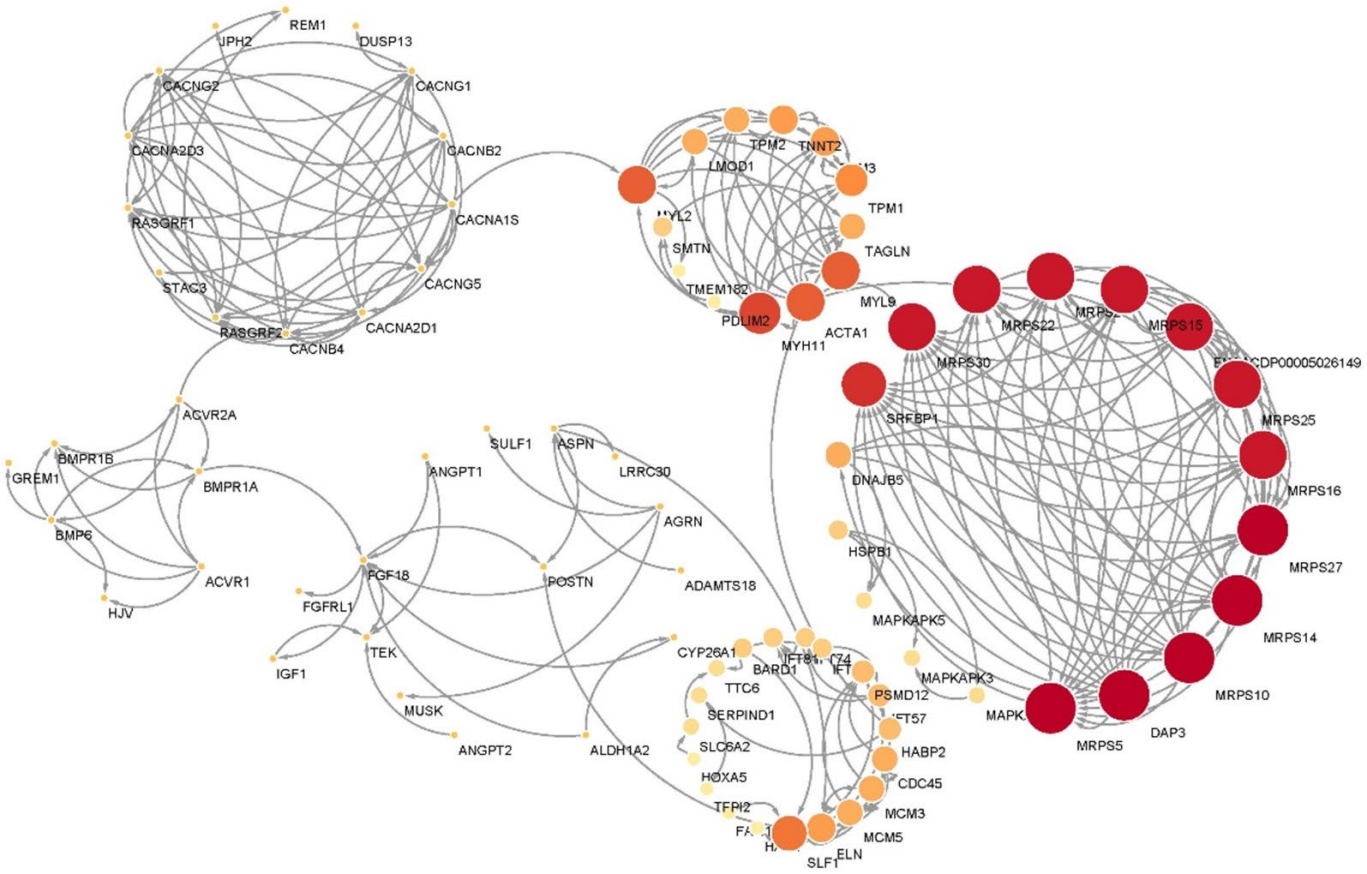
Protein–protein interaction (PPI) network constructed by the STRING web server using DEGs detected in pituitary samples. Node size indicates the degree of each gene.

The upregulated DEGs in pituitary samples were used to construct a protein–protein interaction network. Six clear gene hubs were formed, with biological functions related to myosin development, mitochondrial respiratory chain, calcium metabolism, bone morphogenesis, and *IGF1*-involved growth processes (Fig. 5). The sub-network associated with *HOXA5* was also observed in the analysis.

### Validation of DEGs by qPCR

**Figure 6:**
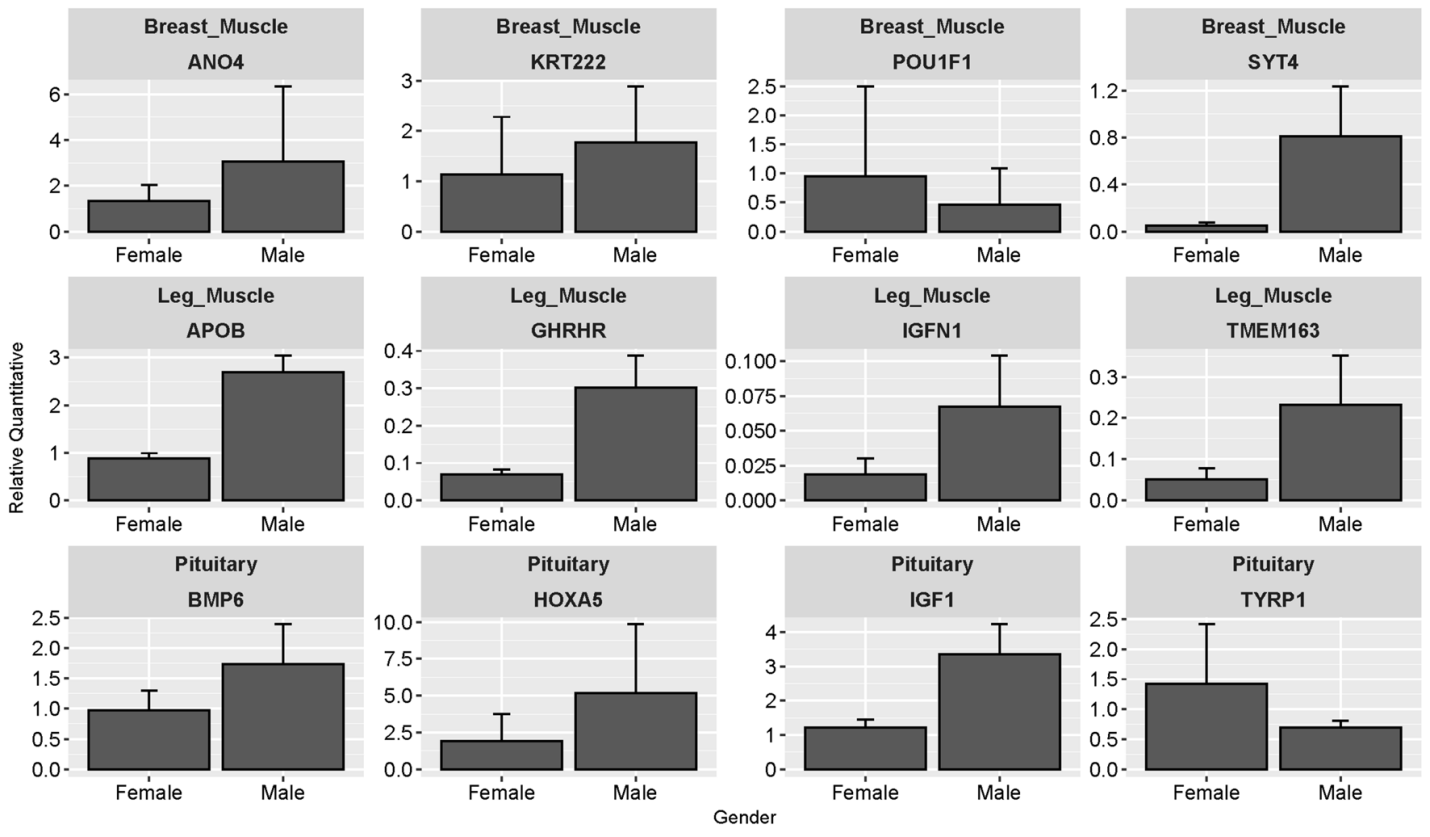
Relative quantitative values for twelve selected DEGs. Expression profiles in leg muscle (panel up) and pituitary (panel down) tissues were tested, while breast muscle tissue was not selected. POU1F1 showed no significantly difference between male and female geese (*P* > 0.05) while expression difference between genders for other genes among tissues showed statistical significance (*P* < 0.05).

For both leg muscle and pituitary tissues, four genes belong to DEGs were randomly selected for qPCR to verify their expression profiles. In breast muscles, upregulated genes (*ANO4*, *KRT222*, and *SYT4*) and downregulated gene (*POU1F1*) were detected with same expression patterns found in our transcriptome sequencing experiments. The expression levels of *POU1F1* in females was higher than males although it showed no statistical significance (top panel in Fig. 5). The significantly upregulated DEGs (*APOB*, *GHRHR*, *TMEM163*, and *IGFN1*) in male leg muscles showed similar expression differences between males and females (Fig. 6). In pituitary tissues, four genes of *HOXA5*, *BMP6*, *TYRP1*, and *IGF1* were all significantly upregulated in male pituitary tissues (Table S1 and S3). Their expression patterns were consistent with results from our RNA-Seq experiments, confirming the reliability of our transcriptome analyses.

## Discussion

### Differences in male and female geese emerged at early developmental stages

In this study, the substantial differences in body weights between male and female ZDW geese prompted us to investigate the underlying mechanisms of gender effects on growth performance (Table 1). Although the selected geese did not show complete sexual maturity, we could identify critical genes and pathways, as males and females had already displayed obvious differences at the macro (Table 1) as well as theo-level micro-level (Fig. 1). The significant difference in weekly body weights between male and female geese persisted during all investigated intervals, and the differences became more apparent with age (Table 1). A further interesting finding was that the concentrations of hormones and the four blood biochemical indices were similar between male and female geese (Tables 2, 3). This could be partially attributed to the young age of the sampled geese. However, it may also indicate that hormones were not the vital factors contributing to differences in growth before sexual maturity during the early developmental stages. Myogenesis is a complicated process that is achieved in several critical steps during different developmental stages, such as embryonic, fetal, perinatal, and postnatal stages^26^. Moreover, several genes, including *PAX3*, *PAX7*, and *MYF5*, all of which belong to the myogenic regulatory factor family (MRF), have been demonstrated to play roles in myogenesis^27–29^. These genes function during very early developmental stages in mice (*e.g.,* E8) and control the differentiation and specification of muscle fibers^30^. Other loci discussed below also supported this finding.

**Table 2:** Concentrations of blood biochemistry index (BCI) in serum among ZDW geese.

Genders	GLU (mmol/L)*	TG (mmol/L)	CHO (mmol/L)	HDL (mmol/L)	LDL (mmol/L)
Male	8.90 ± 1.49^a^	1.20 ± 0.57	5.06 ± 1.17	3.03 ± 0.77	1.57 ± 0.34
Female	7.11 ± 0.85^b^	0.95 ± 0.12	5.53 ± 0.56	3.50 ± 0.28	1.58 ± 0.22

*Different lowercase letters indicated significant difference at the level of 0.05.

**Table 3:** Concentrations of hormones in serum among ZDW geese.

Genders	E2 (pg/mL)	T (pg/mL)	GH (ng/mL)
Male	349.52 ± 10.19	251.14 ± 6.01	14.70 ± 0.53
Female	360.71 ± 11.94	250.67 ± 18.63	14.38 ± 1.72

### Key genes contributed to the difference in growth performance between males and females before hormones exerted effects

Using the upregulated DEGs in male geese, we identified the GO term for hormone activity, which was found in the transcriptomes of various tissues (Fig. 2A, Tables S4 and S6). *IGF1* (pituitary) and *GHRHR* (leg muscle) were among the genes included in this GO term. These genes have been associated with height^31^ and body weight^32,33^. Higher expression levels of these genes were also detected in male geese, potentially contributing to higher weekly body weights in males. However, in all tissues, there were no significant differences between male and female geese for the *GH* gene, a downstream target gene of *GHRHR*. The serum concentrations of GH were in line with this expression pattern (Table 3). This further confirmed that hormone-based pathways were not the sole regulators of growth and development. This was highlighted by the higher expression of the *NCAPG* gene in both the leg and breast muscles of males (Tables S1, S2). *NCAPG* and *LCORL* formed the *NCAPG*-*LCORL* locus, with both being frequently and widely reported for their substantial effects on body size variation in horses^34^, geese^12^, and dogs^35^.

### Multiple effects of gender on the growth and development of male and female geese

While summarizing the precise and systematic effects of sex on goose growth is challenging, our study provides limited but valuable insights into understanding how gender influences growth performance.

First, we identified key DEGs (*TNNT-2*, *MYL-9*, and *MYH-11*) and subsequently enriched the GO terms related to muscle development and muscle fiber architecture, including the sarcomere and its organization*.* Several myosin-light (MYL) and myosin-heavy (MYH) chain genes, upregulated in male geese, were part of this GO term. Certain mutated MYH isoforms have been associated with familial hypertrophic cardiomyopathy (FHC) in humans^36^, indicating their role in muscle development. KEGG pathway analysis also showed significant enrichment for hypertrophic cardiomyopathy, dilated cardiomyopathy, and myocardial contractile disease. Based on this information, we hypothesize that gender may lead to variations in muscle fiber structure. Upregulated DEGs in males would ultimately help develop thicker fibers and a higher muscle mass^37^. Moreover, several DEGs related to calcium metabolism were detected, confirming the unique role of calcium ions in the development of myosin in muscles (Fig. 4). The mitochondrial respiratory proteins have been rarely reported in the last few decades of research to decipher the effects of gender on growth. Two of these genes, *MRPS22* and *MRPS25*, are implicated in human hypertrophic cardiomyopathy and encephalopathy, although the exact functions of this gene cluster in muscle development need further study^38,39^.

We hypothesize that male geese have advantages in nutrient utilization through specific pathways involved in protein and vitamin metabolism (Fig. 2). The beneficial role of vitamins in calcium absorption and growth has long been recognized^40–42^. Moreover, significantly enriched KEGG pathways related to gastric acid and bile secretion suggest a higher feed utilization efficiency in male geese, which is alsoreported in commercial pigs and broilers^7,43^. DEGs (*SORCS3* and *PTPRN2*) associated with feed efficiency in chickens were demonstrated to be significantly upregulated in males^44^. This information reminds us that the accumulation of slim advantages may ultimately enhance feed efficiency in males. Finally, the effect of gender also extends to bone development, as related GO terms, including bone development (leg and pituitary), bone morphogenesis (leg and pituitary), and cartilage development (pituitary), were significantly enriched*.* Key regulators *BMP6* and *TGFB1,* associated with bone and articular cartilage homeostasis^45^, were significantly upregulated in this study. Knock-out experiments in zebrafish have analyzed the functions of *BMP6* in bone development^46^, while *TGFB1* has also been reported to affect bone mineral density in humans^47^. Since key GOs associated with bone mineralization were detected in both the hypophysis and leg musculature (Tables S1 and S3), better growth performance of male geese can be attributed to a higher efficiency in nutrient utilization.

## Conclusions

Our comparative transcriptome analysis between male and female geese has provided novel insights into the mechanisms underlying the substantial differences in growth performance between the sexes. Higher transcriptional activities of critical genes, especially those for *IGF1*, *GHRHR*, and *NCAPG*-*LCORL*, which are related to growth and development, *MRPS22*/*25*, which is related to mitochondrial respiration, and *SORCSS* and *PRPRN2*, which are associated with feed efficiency, were detected in male geese. All DEGs helped us identify important GOs (hormone activity and muscle fiber architecture) and pathways or networks related to cardiovascular development (hypertrophic cardiomyopathy and dilated cardiomyopathy) and calcium metabolism (MARPS gene family). These genes and gene networks demonstrate that not only do many other factors play key roles in the differences in growth performance between male and female geese before complete sexual maturity, but also that there exists a complicated synergistic effect among all these candidates. Nevertheless, ongoing research, especially on nutrient digestion and uptake, continues to improve our understanding.

### Supplementary Information

Supplementary Tables.

## Data Availability

Sequence data that support the findings of this study have been deposited in the NCBI with the project number PRJNA1078079 (https://dataview.ncbi.nlm.nih.gov/object/PRJNA1078079).
